# AbsorbaTack^™^ vs. ProTack^™^ vs. sutures: a biomechanical analysis of cervical fixation methods for laparoscopic apical fixations in the porcine model

**DOI:** 10.1007/s00404-022-06827-3

**Published:** 2022-11-20

**Authors:** Ludwig Sebastian, Jansen Alina, Thangarajah Fabinshy, Ratiu Dominik, Sauerwald Axel, Hachenberg Jens, Wegmann Kilian, Rudroff Claudia, Karapanos Leonidas, Radosa Julia, Trageser Nadja, Eichler Christian

**Affiliations:** 1 Department of Gynecology and Obstetrics, Faculty of Medicine and University Hospital Cologne, University of Cologne, Kerpenerstrasse 34, 50931 Cologne, Germany; 2 Department of Gynecology and Obstetrics, St. Marien Hospital Düren, Düren, Germany; 3 Department of Gynecology and Obstetrics, Hannover Medical School, Hannover, Germany; 4 Department for Trauma, Hand and Elbow Surgery, Faculty of Medicine and University Hospital Cologne, University of Cologne, Cologne, Germany; 5 Department of General Surgery, Evangelisches Krankenhaus Köln-Weyertal, Cologne, Germany; 6 Department of Urology, Uro-Oncology, Faculty of Medicine and University Hospital Cologne, Robot- Assisted and Reconstructive Surgery, University of Cologne, Cologne, Germany; 7 Department for Gynecology, Obstetrics and Reproductive Medicine, Saarland University Hospital, Homburg, Germany; 8 Breast Cancer Center, St. Franziskus-Hospital Münster, 48145 Münster, Germany

**Keywords:** Pelvic organ prolapse, Apical fixation, Cervicosacropexy, Polyvinylidene-fluoride, Uterosacral ligaments, Mesh fixation, Biomechanical analysis

## Abstract

**Purpose:**

Treatment of pelvic organ prolapse (POP) often requires the use of synthetic mesh. In case of a novel and standardized bilateral apical fixation, both uterosacral ligaments are replaced by polyvinylidene-fluoride (PVDF) tapes. One of the main problems remains the fixation method, which should be stable, but also simple and quick to use. The current study evaluated biomechanical differences between the cervical tape fixation with sutures (group 1), non-absorbable tacks (group 2) and absorbable tacks (group 3) in an in vitro porcine model.

**Methods:**

A total of 28 trials, conducted in three groups, were performed on porcine, fresh cadaver uteri. All trials were performed until mesh, tissue or fixation device failure occurred. Primary endpoints were the biomechanical properties maximum load (N), displacement at failure (mm) and stiffness (N/mm). The failure mode was a secondary endpoint.

**Results:**

There was a significant difference between all three groups concerning the maximum load. Group 1 (sutures) supported a maximum load of 64 ± 15 N, group 2 (non-absorbable tacks) yielded 41 ± 10 N and group 3 (absorbable tacks) achieved 15 ± 8 N.

The most common failure mode was a mesh failure for group 1 and 2 and a fixation device failure for group 3.

**Conclusion:**

The PVDF-tape fixation with sutures supports 1.5 times the load that is supported by non-absorbable tacks and 4.2 times the load that is supported by absorbable tacks. Nevertheless, there was also a stable fixation through tacks. Sutures are the significantly stronger and cheaper fixation device but may prolong the surgical time in contrast to the use of tacks.

## What does this study adds to the clinical work

**Table:** 

In the porcine model, suture fixation was shown to be the simplest and most effective technique to fix a PVDF-mesh to the cervix. These findings may contribute to the prevention of POP recurrence in human as well.	

## Introduction

An advancing age is associated with the increasing incidence for pelvic organ prolapse (POP) [1]. More than 19% of women undergo POP surgery in their lifetime and more than 30% of postmenopausal women are affected by POP [2, 3].

Several risk factors for the occurrence of pelvic floor prolapse were examined and the greatest impact besides aging on POP was shown in an increasing parity, waist circumference [2] and genetics [4]. Further problems like urinary or fecal incontinence, voiding or sexual dysfunction may follow [5]. Several treatment options exist, including conservative, like behavioral, physiotherapy or pessary use [6]. If these conservative treatment options fail or in case of advanced POP, surgical treatment options with the use of synthetic mesh are indicated. Because of the importance of apical suspension, mesh sacrocolpopexy is performed as a standard surgical procedure [7].

Ulmsten, Petros and DeLancey emphasized the importance of the holding apparatus of the uterus and vagina, especially the uterosacral ligaments (USL) for apical support and urinary continence [8, 9]. A new surgical technique was developed to achieve the most anatomically accurate and standardized reconstruction of the apical suspension [10–12].

The novel surgical technique, called cervicosacropexy (CESA) and vaginosacropexy (VASA, in case of vaginal vault) was introduced, in which the USL were bilaterally replaced by a minimum of alloplastic material (only 16 cm^2^ mesh used). The surgical technique was “standardized” using polyvinylidene-fluoride (PVDF) tapes of a defined identical length and width, and clearly defined anatomical fixation sides [10, 11].

PVDF meshes show a reduction of mesh-related side effects, as PVDF goes along with a great bio-stability, a low bending stiffness and a minimum tissue reaction with low inflammation parameters. Therefore, PVDF was shown as a possible alternative to commonly used polypropylene (PP) meshes in hernia repair [13, 14].

For CESA, the PVDF tapes with a length of 8.8 cm and a width of 0.4 cm were placed in the peritoneal fold of the USL bilaterally. After supracervical hysterectomy, the tape was attached to the cervical stump with four non-absorbable sutures and was attached to the prevertebral fascia at the level of S1/S2 with two non-absorbable sutures. For VASA, the PVDF tapes have a length of 9.3 cm and are sutured to the vaginal stump [11].

Due to the standardization, the CESA and VASA surgical techniques were adapted into laparoscopic approaches [15–17]. Comparable results could be achieved by laparoscopic cervicosacropexy (laCESA) and laparoscopic vaginosacropexy (laVASA) regarding POP and urinary incontinence.

**Fig. 1:**
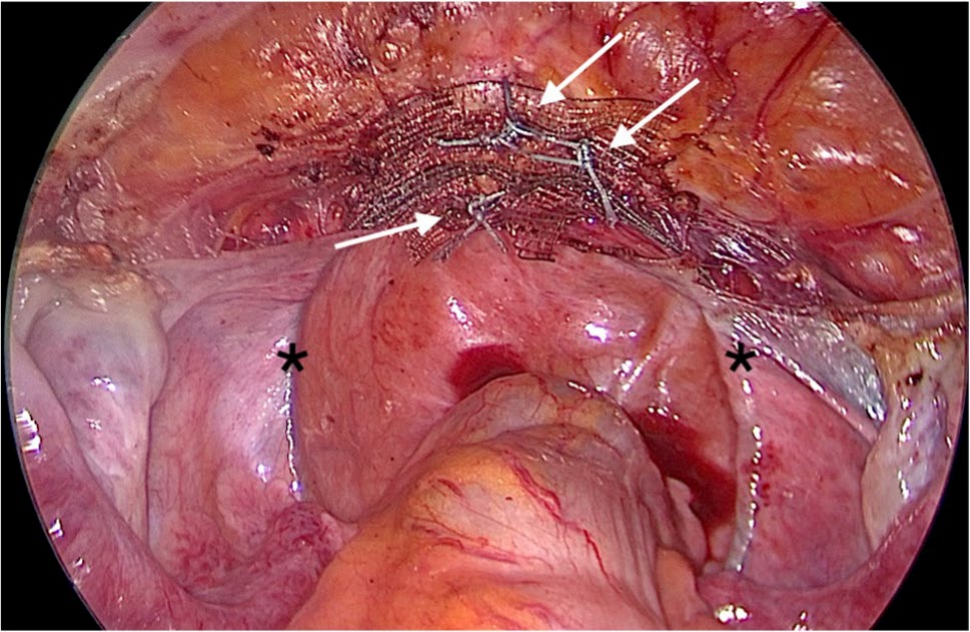
Intraoperative cervical fixation. Cervical fixation of the central part of the PVDF ligament-replacement structure (DynaMesh CESA, FEG Textiltechnik mbH Aachen, Germany) with three non-absorbable sutures (white arrows), PremiCron (HR26s, braided, coated, non-absorbable sutures, Braun Surgical, S.A. Rubi. Spain). The two black asterisks mark each arm of the PVDF-replacement structure for USL replacement. Note that both parts of the PVDF structure already run below the peritoneal fold of the left and right USL

In contrast to Jäger et al. [11] and other study groups [18, 19] in laparoscopic approach, three instead of four interrupted sutures were used to fix the central part of the PVDF-tape on the cervix or the vaginal vault (Fig. 1). For the fixation at the sacrum, three titanium tacks were used [15].

Since laparoscopic suturing demands extensive training and prolongs surgical time [20], the aim of this study was to investigate the main biomechanical properties for the cervical fixation with three interrupted sutures (standard) and to evaluate if a fixation with three non-absorbable titanium tacks, on the one hand, or a fixation device with three absorbable tacks, on the other hand, represents an appropriate alternative to sutures.

Therefore, for the first time in literature, this study explores and compares the biomechanical properties of the three single sutures (standard) with three ProTack^™^ tacks and the three AbsorbaTack^™^ tacks, all in combination with the PVDF tape.

Moreover, the limiting factor (mesh failure, fixation device failure or tissue failure) of the different fixation methods should be evaluated for all groups.

## Methods

The biomechanical in vitro testing was performed on porcine, non- embalmed, fresh and unfrozen cadaver uteri. 28 porcine uteri, divided into three subgroups, were used for the experiments. The uteri were procured from a slaughterhouse in Wachtendonk, Germany and have food grade quality. Each cadaver uterus was only used in one trial. All uteri were prepared and used for the experiments on the same day. Three types of trials were generated. Each group evaluated the cervical tape fixation with a different fixation device: Group 1 (*n* = 10) evaluated three interrupted sutures, group 2 (*n* = 10) three titanium tacks (ProTack^™^, Covidien, Mansfield, MA) and group 3 (*n* = 8) three absorbable tacks (AbsorbaTack^™^ Covidien, Mansfield, MA).

Group 1 used a polyester, braided, coated, non-absorbable PremiCron^®^ suture 1, HR26s needle, 75 cm green filament (B. BRAUN Surgical, S.A. Rubi. Spain).

**Fig. 2:**
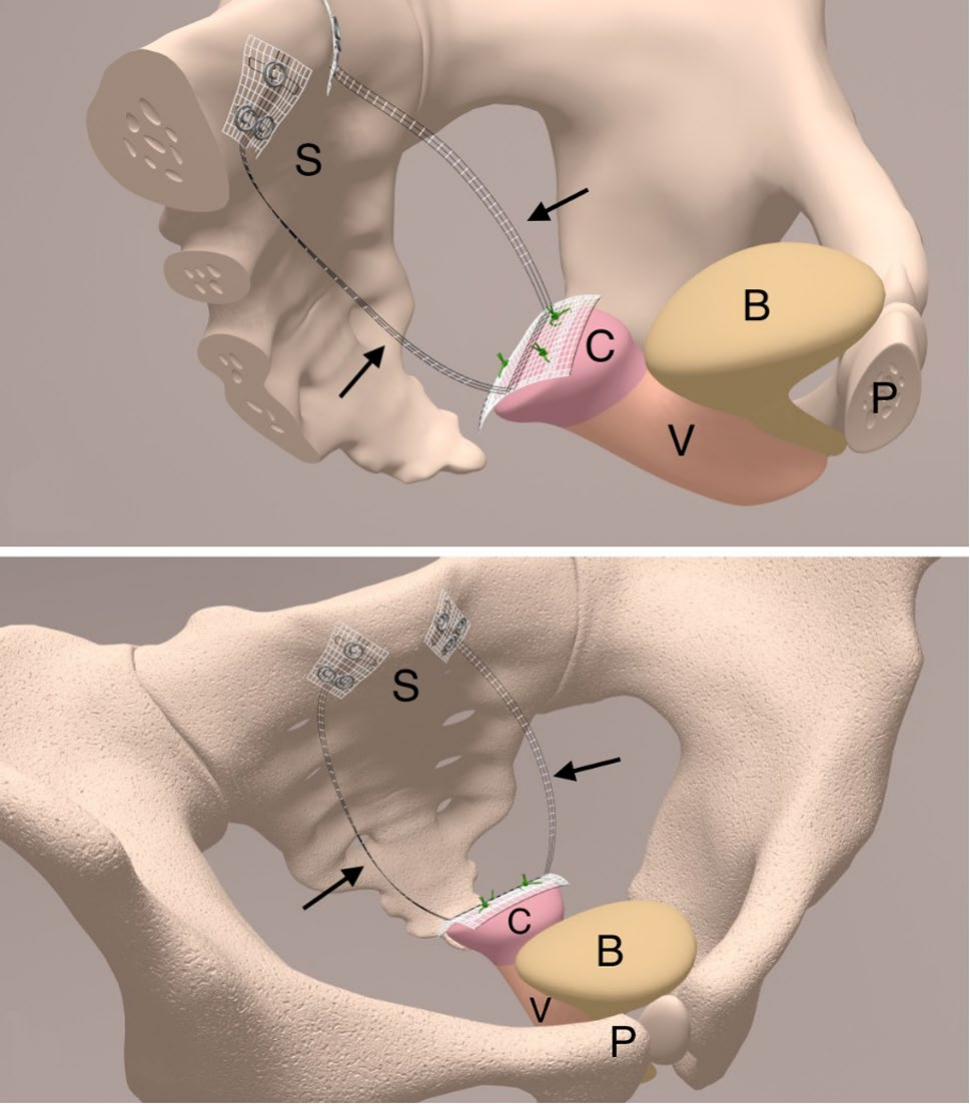
Schematic view of the PVDF-tape in the small pelvis. Position of the specially designed polyvinylidene-fluoride (PVDF) tape (DynaMesh CESA, FEG Textiltechnik mbH Aachen, Germany) for cervicosacropexy (CESA) in the small pelvis. These tapes were attached distally on the cervical stump and proximal to the prevertebral fascia on the sacral vertebra in the level of S1/S2. The two black arrows show the arms of the PVDF-tape, which are intended for bilateral uterosacral ligament (USL) replacement. *S* sacrum; *C* cervix, *V* vagina, *B* bladder, *P* pubic bone

The utilized mesh, previously described as tape, shows a central part (3 × 4 cm) for the fixation at the anterior cervix and two 1 × 2 cm fixation sides for the fixation at the left and right prevertebral fascia of S1/S2 sacral vertebra. Both structures are connected by two 8.8 cm long and 0.4 cm wide arms, which are intended to replace the uterosacral ligaments (USL) bilaterally (Fig. 2). The tapes are composed of non-absorbable, bio-stable polyvinylidene-fluoride (PVDF) monofilaments (DynaMesh^®^ CESA- 03 cm × 04 cm- FEG Textiltechnik mbH Company, Aachen, Germany).

**Fig. 3:**
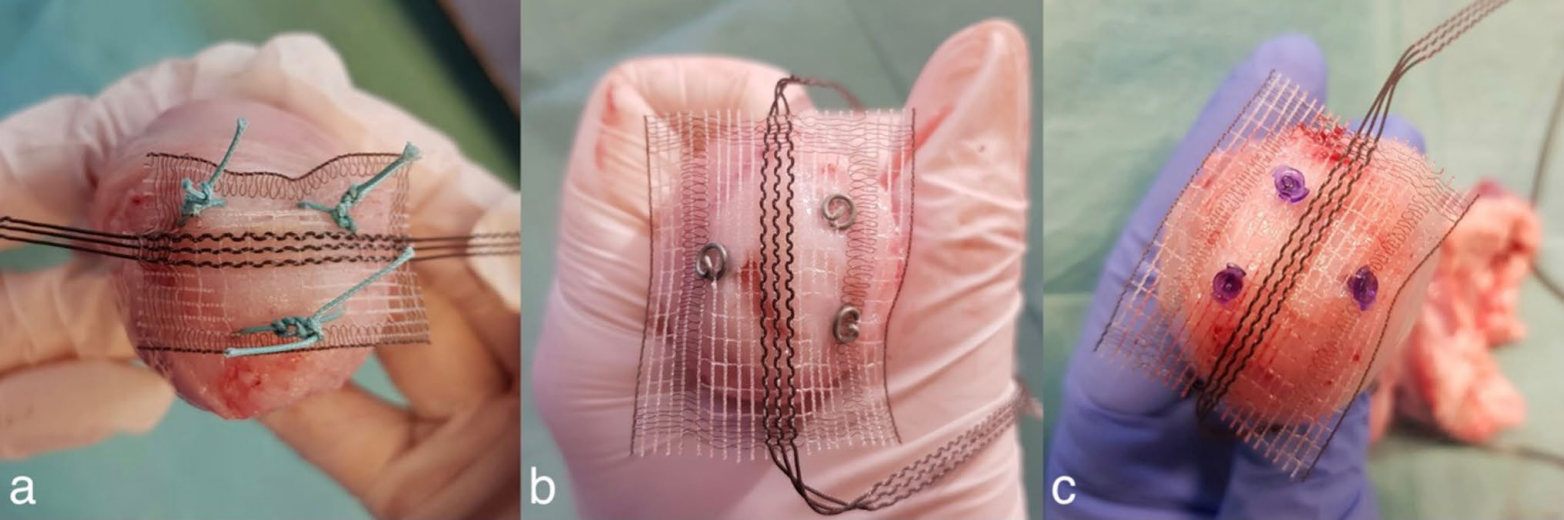
Overview of the different fixation methods. Fixation of the central part of the PVDF-tape (DynaMesh CESA, FEG Textiltechnik mbH Aachen, Germany) with three different fixation devices. **a** Group 1 (three interrupted sutures) used PremiCron (HR26s, braided, coated, non-absorbable sutures, Braun Surgical, S.A. Rubi. Spain). **b** Group 2 (three titanium tacks) used a fixation device (ProTack, Auto Suture 5 mm, Covidien, Mansfield, MA, USA) **c** Group 3 (three violet absorbable tacks) used a different fixation device (AbsorbaTack^™^ Covidien, Mansfield, MA) (ProTack^™^)

Figure 3 illustrates the cervical tape fixation for all three groups with the above described fixation devices. Recorded parameters in all trials were maximum load (N), displacement at failure (mm) and stiffness (N/mm) as primary endpoints.

The maximum load was determined using the displacement–force diagram and defines the point of the highest load in Newton that the cervical tape fixation could withstand.

**Fig. 4:**
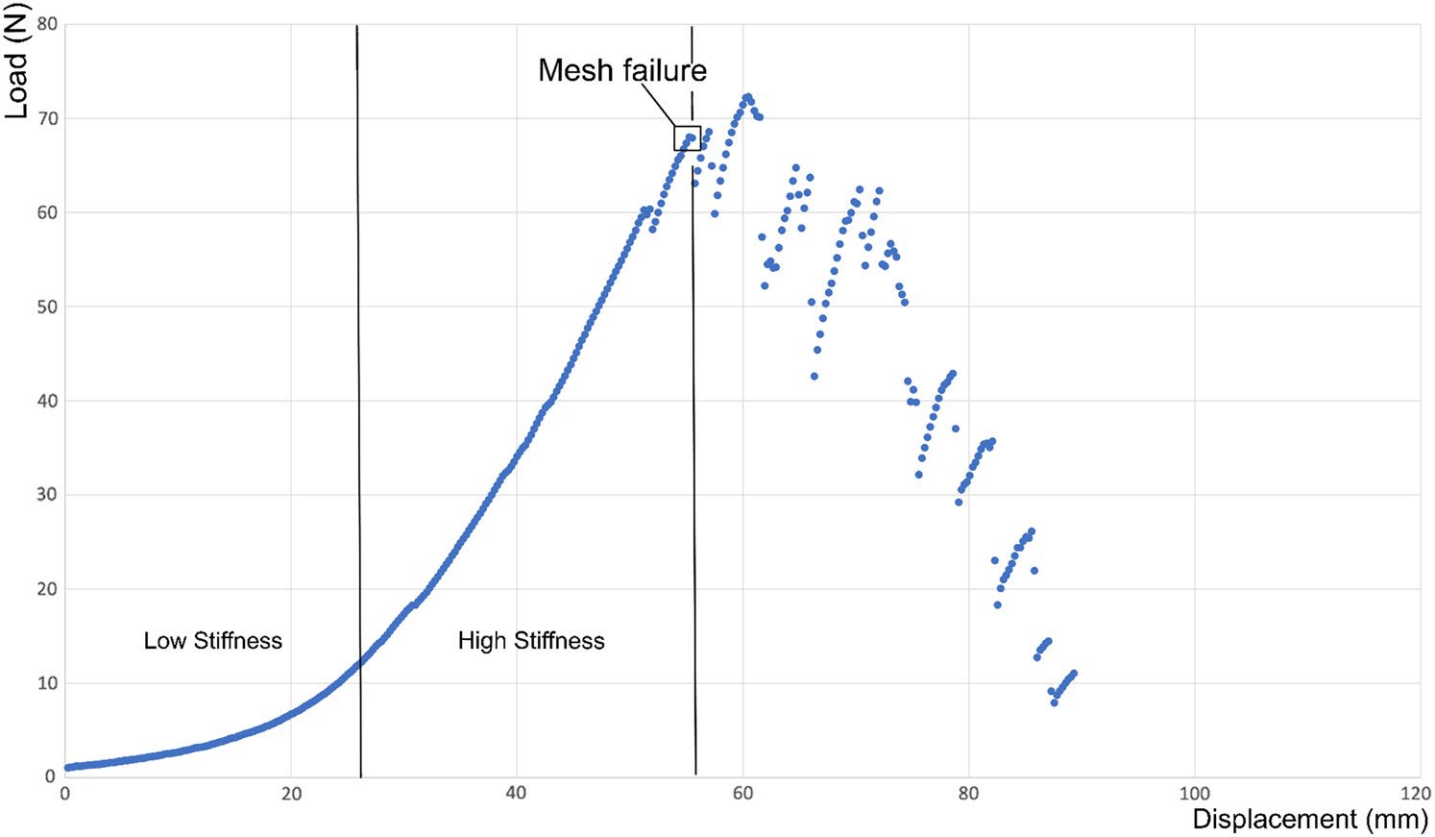
Displacement–force diagram. Shown is a representative displacement–force diagram, exemplary for the tape fixation with sutures (Group 1). On the *Y*-axis, the Load is recorded in Newton (N), on the *X*-axis the Displacement is recorded in mm. At the beginning, the diagram has a part with low stiffness which changes to a part with high stiffness. In this work, stiffness was calculated as the slope in the part with high stiffness. The black box shows the maximum load that the cervical tape fixation could withstand until a mesh failure occurred

The displacement at failure was defined as the elongation of the construct in millimeter (mm) up to the point where the maximum load was reached. The parameter stiffness describes the elongation due to the acting force in Newton (N) and was calculated as the slope out of the displacement-force diagram (Fig. 4). The stiffness varies depending on which point of the diagram it is calculated. At the beginning, there is a part with low stiffness with a fluent transition to a part with higher stiffness [21]. In this work, stiffness was determined in the part with the higher stiffness. Biomechanical tissues have non-linear visco-elastic biomechanical properties [22], which can be seen in the displacement-force diagram.

**Fig. 5:**
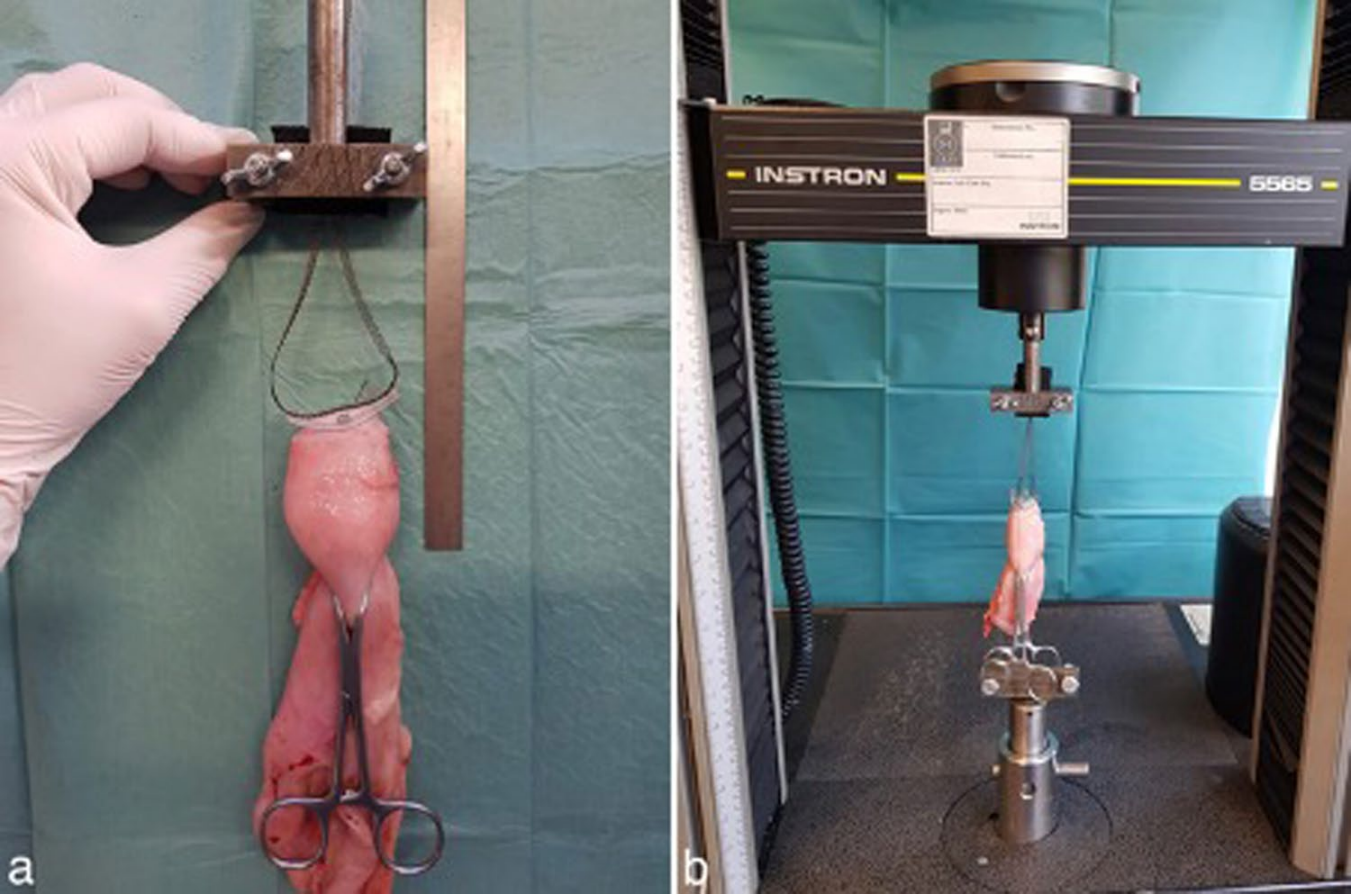
Testing set-up. Shown are representative images of the complete testing set- up for the in vitro experiments with the porcine cervices. **a** There are 5 cm between the surgical clamp and the central part of the PVDF ligament-replacement structure (DynaMesh CESA, FEG Textiltechnik mbH Aachen, Germany) that is fixed on the cervix and 7 cm from this fixation point to the metal bar. **b** Shown is the testing setup for the porcine cervices using the Instron 5565^®^

The failure mode in all trials was evaluated as a secondary endpoint. Analysis was performed on an Instron 5565^®^ test frame using the Bluehill 2 Software^®^. The complete testing set-up is shown in Fig. 5. There are 5 cm between the surgical clamp and the tape fixation point of the cervix and 7 cm from this fixation point up to the metal bar. The arms (thin parts) of the PVDF-tape, which represent the USL replacement structures, were fastened in the metal bar. All trials were performed until mesh, tissue or fixation device failure occurred.

### Statistical analysis

Statistical analysis was performed using the Vassar Stats^®^ (Vassar College, Poughkeepsie, NY, USA) statistics program. ANOVA analysis was used to evaluate significances when appropriate.

## Results

A total of 28 trials were performed and classified in three subgroups. Group 1 (*n* = 10) represents the standard fixation of the PVDF-tape on the cervix with three non-absorbable interrupted sutures. The fixation devices used in group 2 (three titanium tacks, ProTack™) and group 3 (AbsorbaTack^™^) should be compared with this standard in terms of the maximum load (N), displacement at failure (mm) and stiffness (N/mm). A summary of all results is given in Table 1. The maximum load was 64 ± 15 N for group 1, 41 ± 10 N for group 2 and 15 ± 8 N for group 3. Therefore, there is a significant difference (*P* < 0.01) in maximum load between all groups. Evaluating the parameter displacement at failure, there are values of 51 ± 12 mm for group 1 and 52 ± 10 mm for group 2. There is no significant difference between both groups. In contrast, there is a significant difference in displacement at failure between group 1 and group 3 (27 ± 8 mm), on the one hand (*P* < 0.01), and between group 2 and group 3, on the other hand (*P* < 0.01). The third parameter, stiffness, showed a significant difference between group 1 (1.78 ± 0.49 N/mm) and group 2 (1.17 ± 0.3 N/mm) (*P* < 0.05) and between group 1 and group 3 (0.96 ± 0.46 N/mm) (*P* < 0.01), whereas the difference in stiffness between group 2 and group 3 was nonsignificant.

**Table 1:** Overall results for the three evaluated groups performed on the porcine cervices

Evaluated entity	*n*	Maximum load	Displacement at failure	Stiffness	Failure mode
Total trials = 28		N	mm	N/mm	
Group 1 (suture)	10	64 ± 15	51 ± 12	1.78 ± 0.49	Mesh failure (8/10), tissue failure (2/10)
Group 2 (ProTack^™^)	10	41 ± 10	52 ± 10	1.17 ± 0.3	Mesh failure (6/10), fixation device failure (4/10)
Group 3 (AbsorbaTack^™^)	8	15 ± 8	27 ± 8	0.96 ± 0.46	Fixation device failure (8/8)

Overall results for the three evaluated groups performed on the porcine cervices. Group 1 (suture) used PremiCron (HR26s, braided, coated, non-absorbable sutures, Braun Surgical, S.A. Rubi. Spain), group 2 (ProTack^™^) used a fixation device (ProTack, auto suture 5 mm, Covidien, Mansfield, MA, USA) and group 3 (AbsorbaTack™) used a different fixation device (AbsorbaTack™ Covidien, Mansfield, MA) for the fixation of the PVDF (polyvinylidene-fluoride) tapes (DynaMesh CESA, FEG Textiltechnik mbH Aachen, Germany)

*N* newton, *mm* millimeter

**Fig. 6:**
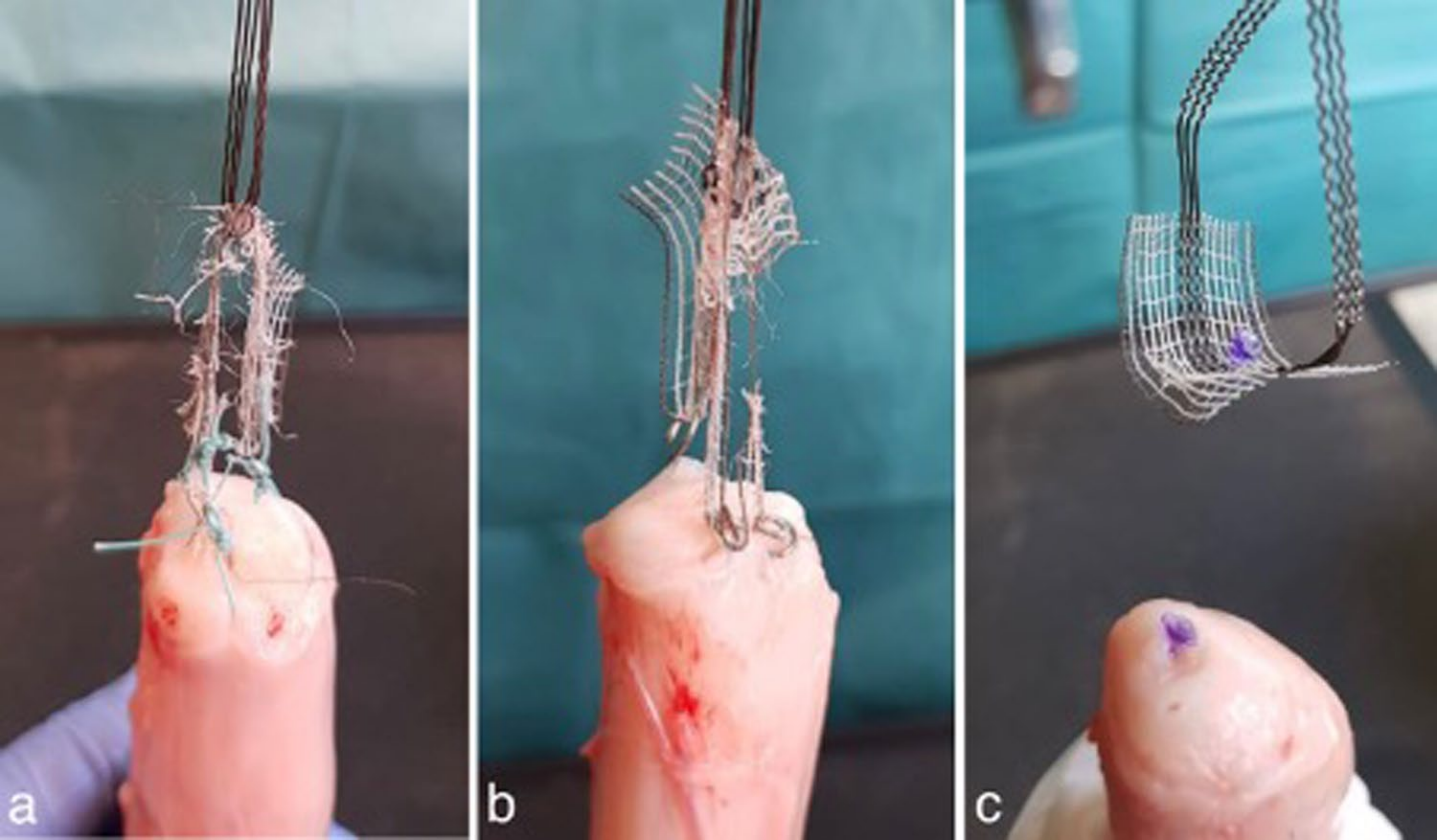
Failure modes for all three groups. Shown are the most common failure modes for the three different fixation devices used with the PVDF-tape (DynaMesh CESA, FEG Textiltechnik mbH Aachen, Germany) on the porcine cervices after the experiments have been performed. **a** Mesh failure in group 1 after the fixation with three single sutures, PremiCron (HR26s, braided, coated, non-absorbable sutures, Braun Surgical, S.A. Rubi. Spain). **b** Mesh failure in group 2 after the fixation with three titanium tacks (ProTack, Auto Suture 5 mm, Covidien, Mansfield, MA, USA). **c** Fixation Device Failure in group 3 after the fixation with three absorbable tacks (AbsorbaTack^™^ Covidien, Mansfield, MA)

The most common limiting factor i.e., failure mode for group 1 (8/10 trials) was a mesh failure, whereby the PVDF tape ruptured as it is shown in Fig. 6a. The sutures were still intact, but due to the acting force from above, the PVDF tape was divided into two parts. A tissue failure occurred in two of the 10 trials. In those cases, the cervical tissue ruptured, which led to a suture tearing.

For group 2 in 6 of 10 trials, there was a mesh failure as well (Fig. 6b). In the remaining 4 of 10 trials, a fixation device failure occurred. The titanium tacks were either loosened completely or bent up in a way the tape was not fixed anymore.

In group 3 occurred a fixation device failure in all cases. In six cases, the absorbable tacks detached one by one from the cervix, and in two trials, they all came loose at the same time. Figure 6c illustrates this fixation device failure.

## Discussion

In a previous study, Rexhepi et al. developed a laparoscopic surgical technique, called laparoscopic bilateral cervicosacropexy (laCESA) and laparoscopic bilateral vaginosacropexy (laVASA), for patients with an apical prolapse suffering from urinary incontinence [15]. In this laparoscopic approach, the central part of the PVDF-tape was fixed on the cervix using three interrupted sutures. In the first four patients, absorbable sutures were used, which led to an insufficient cervical fixation and a relapse of apical prolapse. Subsequently non-absorbable sutures were used instead of the absorbable ones, which did not lead to any apical prolapse [15].

The mean surgical time was 88 min with a range of 34–194 min. Since laparoscopic suturing demands extensive training and since tacks seemed to be the fastest and most easy-to-use method for tape fixation [20], the current study had the aim to compare the biomechanical properties obtained in all three groups and to evaluate, if the cervical tape fixation with absorbable or non-absorbable tacks is an adequate alternative to sutures, which represent the gold standard.

Jansen et al. already provided a biomechanical analysis of sacral bony fixation methods of PVDF-tapes in the porcine model and compared the pre-sacral fixation with sutures with the use of titanium tacks in two different formations. Sutures represent the significantly stronger fixation method since they endure 2.6 times more load than tacks arranged in a row and 1.7 times more load than tacks arranged in a triangle formation [23]. But so far, there has not been any further biomechanical analysis performed in this specific area and there are no data available, comparing sutures with tacks for cervical fixation of these PVDF-tapes for laparoscopic bilateral apical fixation.

There are biomechanical studies performed on human cadaver pelvices that compared laparoscopic suturing methods. Sauerwald et al. [24] concluded that a single suture is not inferior to a continuous approach in pectopexy on the ileo-pectinal ligament.

Hachenberg et al. [25] investigated biomechanical parameters for the sacrospinous ligament and the anterior longitudinal ligament and figured out that an orthogonal suture is superior to an in-line suture at the sacrospinous ligament and that a continuous suture is superior to a single suture at the anterior longitudinal ligament. Moreover, there is one study conducted by Formijne Jonkers et al. that compared three different fixation methods in laparoscopic ventral rectopexy. The use of two single sutures was compared to the fixation with three ProTack^®^ tacks, concluding that there is no significant difference between both fixation devices [20]. This study was performed on a spinal column. Due to the different tissue structures, those results are not transferable to the muscular cervical tissue, which is evaluated in the current study. As there is no study in the current literature, which compares the outcome of different fixation devices for cervical fixation with PVDF-tapes, the current study is the first of its kind.

Comparing the results for maximum load, there is a significant difference between all groups. Hence, it can be concluded that the PVDF tape fixation with three non-absorbable sutures (64 ± 15 N) is significantly superior to the PVDF-tape fixation with non-absorbable tacks (41 ± 10 N) and to the fixation with three absorbable tacks (15 ± 8 N) in terms of the maximum load. Non-absorbable sutures supported 1.5 times the load that is supported by non-absorbable tacks and 4.2 times the load that is supported by absorbable tacks.

The most common limiting factor, i.e., failure mode, was a mesh failure for group 1 and group 2, so that the fixation itself supported more load than the PVDF-tape. Literature does not report about any mesh erosion after PVDF-tape fixation with sutures after bilateral cervicosacropexy [15, 18, 19, 26]. So from a clinical point of view, the factor “mesh failure” does not seem to have any influence on the surgical results, because follow-ups four months after surgery showed only a relapse of apical prolapse because of an insufficient cervical fixation, but all further patients had an apical POP-Q stage 0, which means that the right anatomical position could be restored and the mesh was still intact [15]. The only aspect to mention is the fact that in group 2, a fixation device failure in four of ten cases occurred, which means that the tacks were loosened or tore out, which led to an insufficient tape fixation. On the other hand, in 8 of 8 trials, the fixation with three absorbable tacks failed because of a fixation device failure, whereas the tape was still intact. Until now, laCESA was not performed with non-absorbable or absorbable tacks as cervical tape fixation, so there is no clinical experience yet. As it can be seen from the results in group 3, the fixation device with absorbable tacks only lasts values of 15 ± 8 N in average. Since the fact, that in one trial, all tacks have become detached by a load of 6 N, it can be concluded that this fixation device does not represent an appropriate alternative to the standard fixation with interrupted sutures.

Since the mesh fixation technique is one of the most controversially discussed topics and since the use of absorbable and non-absorbable tacks is the most commonly used technique for mesh fixation in ventral hernia repair [27], in current literature, there are several studies comparing the use of absorbable and non-absorbable tacks in laparoscopic hernia repair.

Christoffersen et al. emphasized that the type of mesh fixation is an independent risk factor for recurrence and showed that the use of absorbable tacks is associated with a higher recurrence rate than the use of non-absorbable tacks, whereby there was no significant difference in terms of chronic pain between both groups [28].

In contrast to the above-mentioned study, Cavallaro et al. compared the fixation of a lightweight polypropylene mesh in laparoscopic incisional hernia repair by non-absorbable tacks (ProTack^™^) with the fixation by absorbable tacks (Securastrap^™^) and concluded that absorbable tacks do not increase the risk of a post-operative mesh dislocation in comparison to non-absorbable tacks. A lower post-operative pain and a shorter hospitalization time could be observed in the group that was treated with absorbable tacks [29].

In group 2 and 3 of the present study, the inferiority of the absorbable tacks could be shown in contrast to the non-absorbable tacks, as non-absorbable tacks support 2.7 times the load that is supported by absorbable tacks.

Regarding the different shape of the absorbable tacks, this could be an explanation for these differences in the final results compared to current literature. The tacks used in the current study (AbsorbaTack^™^) are helicoidal and need a spin rotation to penetrate the cervical tissue, whereas the Securastrap^™^ tacks, which are used in the study conducted by Cavallaro et al. [29], are configurated in a “U” shape and are specifically engineered to be used with Physiomesh^™^, which is used in this study for laparoscopic incisional hernia repair. Furthermore, the anatomical location could explain the differences, as the absorbable tacks are attached to the abdominal wall in laparoscopic hernia repair, whereas the tacks are attached to the cervix in the current study.

It would be necessary to perform further studies to evaluate the biomechanical properties of the cervical tape fixation with different absorbable tacks, which are not helicoidal, but have another shape like the “U” shape of the Securastrap^™^ tacks and may therefore support a higher maximum load in combination with the PVDF-tape.

According to the intraoperative measurements (data not published), it takes in average 90 s to place one interrupted suture in laparoscopy, whereas it takes only 10 s to set one tack, no matter if absorbable or non-absorbable tacks. In total, for a cervical fixation with three single sutures, it would occupy 270 s of surgical time. In contrast, for the fixation with three tacks, it would take 30 s, which is nine times faster than laparoscopic suturing. Comparing the costs of the different fixation devices, sutures cost about 10 euros for one piece, whereas the absorbable tacks as well as the non-absorbable tacks cost about 300 euros for one fixation device and are considerably more expensive.

Considering the parameter displacement at failure for all groups, there is no significant difference between group 1 (51 ± 12 mm) and group 2 (52 ± 10 mm), whereas group 3 (27 ± 8 mm) shows a clear inferiority compared to group 1 and group 2. In the current literature Sauerwald et al. [24] described a displacement at failure of 37 ± 12 mm for a single suture in combination with a mesh and 36 ± 5 mm for a continuous suture with a mesh in pectopexy. There is no significant difference between both groups. Hachenberg et al. [25] detected values of 29 mm displacement at failure for a single suture and 42 mm for a continuous suture with a superiority of the continuous approach. Since both studies investigated the difference between a single and a continuous suture, those results cannot be transferred to the current study. Nevertheless, all values are in a similar range of magnitude.

Analyzing the parameter stiffness, the interrupted sutures on the cervix are significantly stiffer than the non-absorbable tacks in group 2, as well as stiffer than the absorbable tacks in group 3. Between group 2 and 3, there is no significant difference.

The biomechanical analysis of different fixation methods for the cervical mesh fixation in laCESA and laVASA yielded the following results:

Three interrupted sutures in combination with the PVDF tape (gold standard) supported a maximum load of 64 N, a displacement at failure of 51 mm and a stiffness of 1.78 N/mm, whereas three ProTack^™^ tacks supported a maximum load of 41 N, a displacement at failure of 52 mm and a stiffness of 1.17 N/mm and three absorbable tacks led to a maximum load of 15 N, a displacement of 27 mm and a stiffness of 0.96 N/mm.

The most common limiting factor i.e., failure mode was a mesh failure for group 1 (8/10 trials) and 2 (6/10 trials). For group 1, in 2/10 trials occurred a tissue failure. In group 2, in 4/10 trials, there was a fixation device failure. The limiting factor in group 3 was a fixation device failure for all trials.

## Conclusion

This study provides the biomechanical analysis with different fixation methods for PVDF-tape fixation at the cervix in case of apical suspension. Further, this study compares biomechanical properties of suture fixation with non-absorbable and absorbable tacks.

Given the data above, it can be concluded that the PVDF-tape fixation with simple sutures (group 1) is superior to the fixation with titanium tacks (group 2), as well as the fixation with absorbable tacks (group 3) in terms of the maximum load. Sutures support 1.5 times the load that is supported by non-absorbable tacks and 4.2 times the load that is supported by absorbable tacks. Despite the significant results for maximum load, the titanium tacks showed sufficient strength for practical use beside to sutures.

The fixation with absorbable tacks partly only withstood a load of 6 N, which corresponds to only a very low load. Therefore, it can be concluded, that this fixation device does not represent an appropriate alternative to sutures or titanium tacks. Sutures are the significantly stronger and cheaper fixation device but may prolong the surgical time in untrained surgical hands in contrast to tacks.

It is always the aim to achieve the shortest possible operating time. Since studies on different fixation methods in laCESA and laVASA are not available yet, this study provides first insights and compares three fixation devices used with the PVDF tapes.

These results help further standardize the surgical method of a bilateral apical fixation, which contributes in favor of reproducibility and thus comparability of results and avoids apical recurrence.

## Data Availability

The datasets generated and analyzed during the current study are available in the OSF repository, https://osf.io/q957x/.

## Code availability

https://doi.org/10.17605/OSF.IO/Q957X.
